# Editorial: Adaptation of halophilic/halotolerant microorganisms and their applications

**DOI:** 10.3389/fmicb.2023.1252921

**Published:** 2023-08-22

**Authors:** Rosa María Martínez-Espinosa, Sumit Kumar, Sudhir K. Upadhyay, Furkan Orhan

**Affiliations:** ^1^Department of Biochemistry, Molecular Biology, Edaphology, and Agricultural Chemistry, Faculty of Sciences, University of Alicante, Alicante, Spain; ^2^Applied Biochemistry Research Group, Multidisciplinary Institute for Environmental Studies “Ramón Margalef” University of Alicante, Alicante, Spain; ^3^Amity Institute of Biotechnology, Amity University, Noida, Uttar Pradesh, India; ^4^Department of Environmental Science, Veer Bahadur Singh Purvanchal University, Jaunpur, Uttar Pradesh, India; ^5^Faculty of Arts and Science, Department of Molecular Biology and Genetics, Ağrı Ibrahim Çeçen University, Ağrı, Türkiye

**Keywords:** halophilic, enzyme, compatible solutes, bioactive compounds, PGP

In hypersaline soils and waters, microorganisms surviving in these ecosystems must deal with excess salt in addition to any other factors limiting survival. Halophilic and halotolerant microorganisms use a variety of strategies to maintain osmotic equilibrium across their cell membranes and prevent the loss of cytoplasmic water. Among these strategies, modifications at molecular levels affecting proteins and RNA/DNA, salt-in adaptation, compatible solute adaptation, and salt-stable cell surface and membranes are included.

Due to their physiological adaptations, halophilic/halotolerant microorganisms have great potential for diverse applications. The Research Topic “*Adaptation of halophilic/ halotolerant microorganisms and their applications*” includes review and original research articles on the uses of halotolerant and halophilic microorganisms in a variety of fields, including agriculture, medicine, pharmaceuticals, industry, food, and waste treatments such as the degradation of hydrocarbons, and saline wastewater treatment.

Halotolerant and halophilic microorganisms have developed versatile molecular mechanisms for coping with saline stress, and many of these molecular adaptations have potential applications in biotechnology. Within this context, Zhou et al. have explored the mechanisms of halotolerance in six type strains of *Pontixanthobacter* and *Allopontixanthobacter* by comparative genome analysis. Genes directly connected to halotolerance include those involved in osmolytes synthesis, membrane permeability control, ions transport, intracellular signaling, polysaccharide biosynthesis, and SOS response. Similar gene content has been described previously in other bacteria, thus reinforcing the idea that these are the main mechanisms explaining halotolerance. The authors are linking genome-wide co-occurrence, genetic diversity, and physiological characteristics of these bacteria.

Metagenomics as a culture-independent tool has also been employed to harness the biotechnological potential of halophiles. On similar lines, Jeilu et al. identified novel carbohydrate-degrading enzymes using functional metagenomic analysis in samples from Ethiopian Soda Lakes. A total of 378 genes mostly belonging to multiple Glycoside Hydrolases (GH) were identified. Most GH genes were of bacterial origin, predominantly of the *Halomonas* genus. Biochemical analysis of amylase, cellulase, and pectinase revealed them to be polyextremophilic with activity at high temperatures, pH, and salt concentrations. Such properties are relevant for enzymatic applications, particularly in lignocellulosic biorefinery.

To explore polysaccharide-hydrolyzing genomic potential of cultured haloarchaea, Elcheninov et al. reported a comparative genomic analysis of 155 haloarchaeal bacterial strains including seven different genera as *Natronolimnobius, Halococcoides, Halosimplex, Natronobiforma, Halomicrobium* and *Natrarchaeobius*. The authors observed an overpresentation of cellulase genes (GH9, GH12, and GH5) in the cellulotrophic haloarchaea genomes compared to cellulotrophic archaea on a per-genome basis. The research findings also indicated variations in CAZymes profiles among the groups (neutrophilic and alkaliphilic haloarchaea), relating to genome size, the number of genes involved in import mechanisms, and central metabolism of sugars.

The study by Tu et al. used cultivation and high-throughput sequencing techniques to investigate the microbial community of Dingyuan Salt Mine, and to study the effects of long-term brine storage on the microbial community. The dominant bacterial species in fresh brine were *Cyanobium* PCC-6307 spp., *Aeromonas* spp. and *Pseudomonas* spp., whereas the dominant archaea were *Natronomonas* spp., *Halapricum* spp., and *Halomicrobium* spp. After 3-year storage, the microbial community shifted toward *Salinibacter* spp. and *Alcanivorax* spp. as dominant bacterial species and *Natronomonas* spp. and *Halorientalis* spp. as archaeal species. Long-term storage of brine resulted in increased biomass but species diversity declined. This study also led to the isolation of 12 possible new species belonging to 3 genera of halophilic archaea.

Halophiles have also been a great repository for valuable bioactive compounds of pharmaceutical importance. In this context, Karthik et al. investigated the potential of mangrove microbe *Glutamicibacter mysorens* for antimicrobial and anticancer properties, and demonstrated anticancer activity of intracellular metabolites on prostate cancer cells. Low molecular weight compounds Kinetin-9- ribose and Embinin were identified by Liquid Chromatography–Mass Spectrometry (LC–MS) study. Thus, *G. mysorens* is a promising source for low molecular weight bioactive molecules with therapeutic potential.

The review of Moopantakath et al. demonstrated the ecology and diversity of haloarchaeal microorganisms, their strategies in coping with stress, haloarchaea biotechnological significance (anticancer compounds, antimicrobial compounds, antioxidant compounds), hydrolytic enzymes, biodegradable and biocompatible polymers, and synthesis and application of bioactive nanoparticles from haloarchaeal microorganisms.

Haloarchaea are a promising group of microorganisms for biotechnological applications, showing metabolic capabilities of interest for industrial processes within the circular economy, for example the biodegradation and use of the two dominant biomass polysaccharides on the planet, cellulose and chitin. Related to polysaccharide biodegradation, Sorokin et al. conducted a selective enrichment on a wide polysaccharide spectrum aiming at the isolation of novel metabolic and taxonomic groups of haloarchaea from hypersaline lakes. By using an array of commercially available homo- and heteropolysaccharides to enrich hydrolytic haloarchaea, the authors isolated a range of halo- and natronoarchaea, including previously described taxa and several new genus-level lineages. This study demonstrates previously unrecognized microbial potential for utilization of a broad range of natural polysaccharides in hypersaline habitats.

One is the major compatible solutes produced by halophiles is ectoine. Using the ectoine-excreting strain *Halomonas elongata* KB2.13, Hobmeier et al. demonstrated two methods of ectoine production, based on Oxaloacetate-enhanced precursor and on over-expression of transporter [transporter for ectoine accumulation (Tea ABC)]. Both techniques have the potential to significantly increase ectoine production and excretion. This increase was initially attributed to the absence of phosphoenolpyruvate carboxykinase, which converts the oxaloacetate (OAA) into Phospoenolpyruvate (PEP), thus removing feedback inhibition and allowing the unconverted OAA to enter the TCA cycle for ectoine production. The excretion rate of ectoine was significantly enhanced three-fold when both TeaBC subunits, a transporter responsible for ectoine uptake, were overexpressed in the absence of the substrate-binding protein TeaA. The main subunit TeaC showed an extracellular ectoine concentration per dry weight that was roughly five times higher than TeaBC shortly after its expression was induced. Since both approaches are complementary, they are promising solutions for metabolic engineering challenges.

In deep shale reservoirs, salinity and hydraulic retention time (HRT) have an impact on *Halanaerobium* cell membrane structure, which in turn affects microbial development and physiology and causes biogeochemical responses. The variations in the membrane fatty acid chemistry of *H. congolense* WG10 caused by salt and HRT have been addressed by Ugwuodo et al.. Notably, *H. congolense* WG10 increases the amount of polyunsaturated fatty acids in its membrane under suboptimal salt concentrations, which appears to increase its fluidity and thickness. Mean chain length and double bond index are used as proxies for the fluidity and thickness of the membrane, respectively. Thus, natural and human-made variables may alter the chemistry of membrane fatty acids in persistent microbial taxa that are important to maintain physical and biogeochemical equilibrium of fractured shale, with implications for human health.

The review article of Ramasamy and Mahawar offers novel perspectives on the role of halotolerant (HT) bacteria linked to crop plants in boosting their resistance to salinity stress. The paper also identifies several issues with halotolerant plant growth promoting Rhizobacteria (HT-PGPR)'s application in the agricultural sector and suggests scientific ways to solve them to advance sustainable agriculture in the future.

Based on research conducted by John et al. the inoculation of *Vigna mungo* L., a legume, with halotolerant plant growth promoting rhizobacteria (HT-PGPR) isolated from *Sesuvium portulacastrum*, led to increased shoot length and vigor index, indicating a potential enhancement in salt tolerance for the plant. Moreover, the salt tolerant bacterial inoculation led to enhancements in grain yield, shoot length, chlorophyll content, and photosynthetic rate, while also reducing the enzymatic activity of catalase and superoxide dismutase, suggesting improved stress tolerance. The research findings also suggest that HT-PGPRs can be a cost-effective and ecologically sustainable approach to enhance crop productivity in high saline conditions. The use of such rhizobacteria holds significant promise for sustainable agricultural practices in salt-affected regions.

In conclusion, most saline ecosystems of our planet are still unexplored for both basic and applied sciences studies. The study of the microbiome of these environments by culture-dependent and -independent techniques will reveal a great deal of microbial diversity. Furthermore, these halophilic microbes can be a biotechnologically useful source of robust enzymes and of pharmaceutical molecules with potential for industry, agriculture, and environmental bioremediation. Research carried out on halophiles to date has established that halophiles can serve as an important tool for biological interventions.

## Author contributions

All authors listed have made a substantial, direct, and intellectual contribution to the work and approved it for publication.

